# Implantation cutaneous tuberculosis after ultrasound-guided fine needle aspiration cytology

**DOI:** 10.1259/bjrcr.20150393

**Published:** 2016-07-28

**Authors:** Usha Dutta, Deepa Shrestha, Amit Sharma, Parikshaa Gupta, Ashim Das, Radhika Srinivasan, Lal Anupam, Vishal Sharma, Surinder Singh Rana

**Affiliations:** ^1^Department of Gastroenterology, Postgraduate Institute of Medical Education and Research, Chandigarh, India; ^2^Department of Histopathology, Postgraduate Institute of Medical Education and Research, Chandigarh, India; ^3^Department of Cytology and Gynaecological Pathology, Postgraduate Institute of Medical Education and Research, Chandigarh, India; ^4^Department of Radiodiagnosis and Imaging, Postgraduate Institute of Medical Education and Research, Chandigarh, India

## Abstract

A 45-year-old female presented with recurrent biliary pain, fever, anorexia and weight loss of 3 months duration. She was on highly active antiretroviral therapy for underlying human immunodeficiency virus infection for the past 5 months. Clinical examination revealed a 3-cm firm hepatomegaly. Investigations suggested mild anaemia, elevated erythrocyte sedimentation rate, deranged liver function tests, Mantoux test of 25 mm and CD4 count of 417 cells μl^−1^. Ultrasonography revealed mild central intrahepatic biliary radical dilatation with a dilated common bile duct and multiple periportal and peripancreatic lymph nodes. Ultrasound-guided fine needle aspiration cytology (FNAC) from the periportal lymph node was performed twice. Both were reported as only bloody aspirate. The patient developed an erythematous tender skin nodule at the site of insertion of the FNAC needle 15 days after the second FNAC procedure. An excision biopsy of the nodule showed ill-formed epithelioid cell granulomas with acid-fast bacilli, indicating tuberculosis. The patient was started on antitubercular therapy with complete response.

## Background

Fine needle aspiration cytology (FNAC) is used for obtaining tissue material for diagnosis of various conditions. It is regarded as an easy, safe, rapid and minimally invasive procedure to establish the diagnosis. Complication rates following FNAC are low. The use of ultrasound guidance has led to further increase in the diagnostic yield and decrease in the complication rates. Guided FNAC also allows access to difficult sites. Minor complications such as pain, infection and bleeding are well described. Major complications, including needle tract seeding, have been reported primarily in patients with malignancy.^1^

Needle tract seeding refers to implantation of the aspirated material along the tract by the instrument used to examine, excise or ablate a lesion. This iatrogenic phenomenon has typically been defined and described in relation to malignant conditions. Aspirates from the liver, thyroid, pancreas, intestine, kidney, breast, lung and pleura are more often associated with this complication.^1^ Alhough the incidence of needle track seeding is considered low (0.005–0.009%), diagnostic aspiration is best avioded if curative resection is being planned in malignant conditions.^2–4^

Seeding is usually considered an unfavourable outcome as it results in dissemination of the tumour, making curative resection difficult. We report the case of a 45-year-old female with abdominal lymph nodal tuberculosis in the setting of human immunodeficiency virus (HIV) infection, in whom FNAC of the involved lymph node resulted in implantation of tubercular bacilli along the needle tract, which resulted in cutaneous tuberculosis.

## Case report

**Figure 1.**
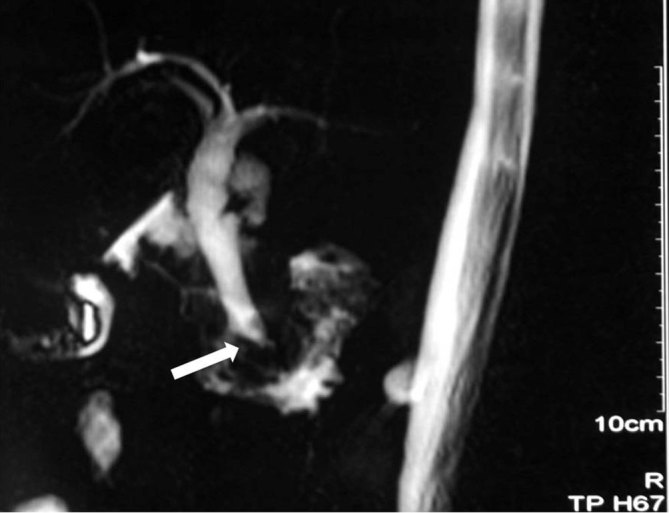
MR cholangiopancreatography showing the dilated common bile duct with abrupt cut-off (arrow).

A 45-year-old female was diagnosed with HIV infection in December 2013. She was started on highly active antiretroviral therapy comprising tenofovir 300 mg once daily (OD), lamivudine 300 mg OD and nevirapine 200 mg twice daily 5 months back, which she was tolerating well. Her CD4 count was 417 cells μl^−1^. She presented to us in June 2014 with pain in the right upper quadrant of 3 months duration that was intermittent, colicky, moderate in intensity and radiating to the infrascapular area, suggesting a biliary origin and requiring i.v. analgesics on multiple occasions. She also had low-grade fever, anorexia and weight loss of the same duration. There was no jaundice or other systemic features. Clinical examination revealed a 3-cm firm hepatomegaly; there was no peripheral lymphadenopathy and the rest of the clinical examination was unremarkable. Blood investigations suggested mild anaemia (Hb 10.2 g dl^−1^), elevated erythrocyte sedimentation rate (47 mm in the firsthour) and deranged liver function tests [serum bilirubin: 0.6 mg dl^−1^; albumin 4.0 g dl^−1^; aspartate aminotransferase 49 U l^−1^; alanine aminotransferase 145 U l^−1^; alkaline phosphatase 460 U l^−1^ (normal 42–128 U l^−1^)]. Ultrasonography of the abdomen showed mild central intrahepatic biliary radical dilatation with a dilated common bile duct (CBD) and multiple periportal and peripancreatic lymph nodes, the largest measuring 10 mm in diameter. Dynamic contrast-enhanced MRI and MR cholangiopancreatography were performed, which revealed a dilated CBD with an abrupt cut-off of the distal CBD with few subcentimetre lymph nodes (Figure 1). Mantoux test was positive with an induration of 25 × 25 mm at 72 h. Ultrasound-guided FNAC from periportal lymph nodes was performed with a 22-gauge spinal needle and two passes were obtained with the same needle on two separate occasions 1 week apart. However, on cytological examination, the aspirate was bloody and no conclusive diagnosis could be offered by the cytopathologist.

**Figure 2.**
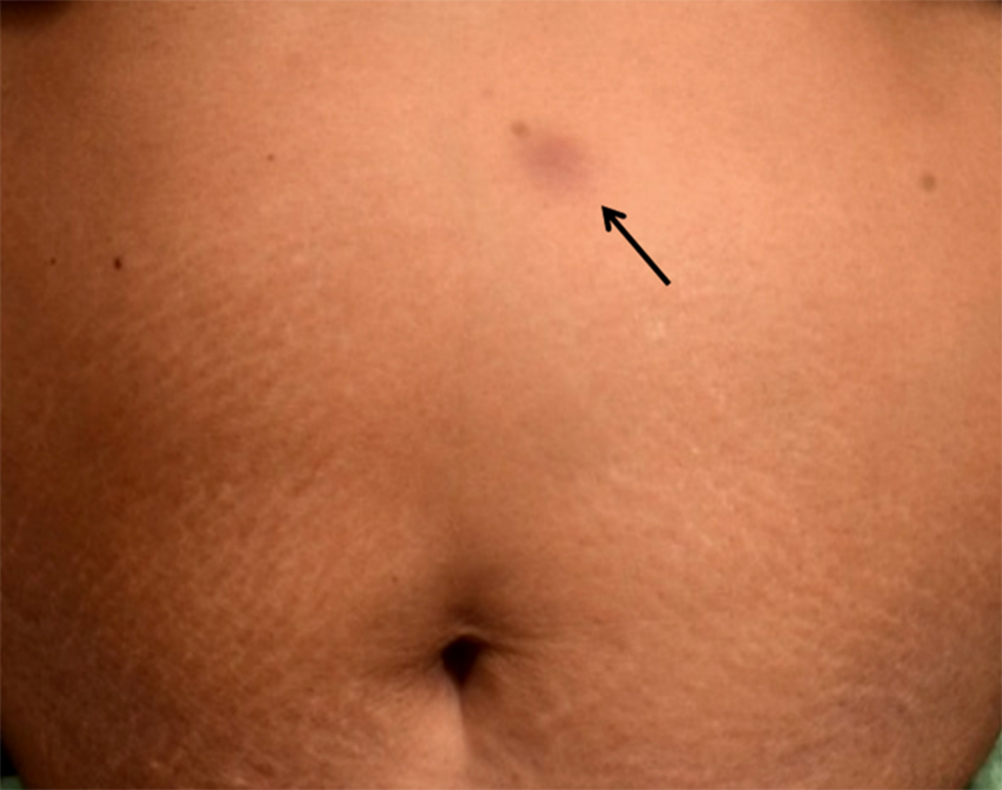
Erythematous, tender, superficial nodule on the skin that developed at the site of fine needle aspiration cytology (arrow).

15 days after the second FNAC, the patient developed a painful skin nodule at the site of insertion of the FNAC needle. The nodule measured 1 × 1 cm and was erythematous, well-defined, firm and mildly tender, as shown in Figure 2 (indicated by arrow). FNAC tract seeding by tuberculosis was suspected and excision biopsy of the nodule was performed.

**Figure 3.**
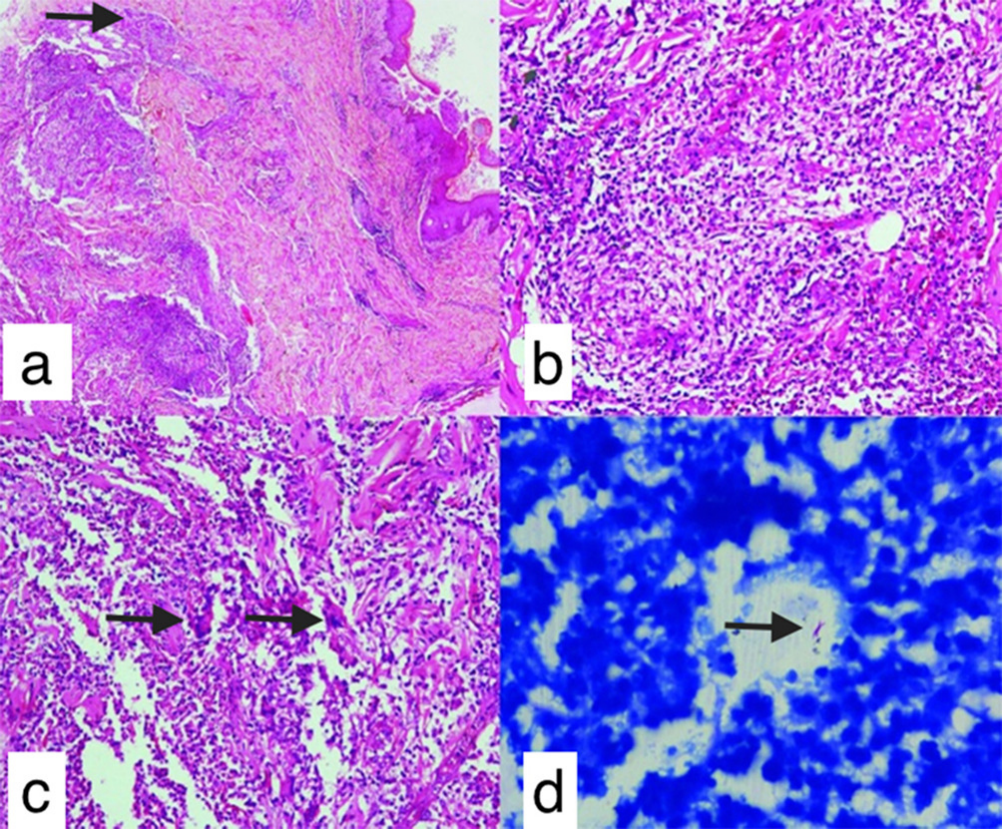
(a) Section showing unremarkable epidermis with moderate to dense perivascular and periappendageal inflammatory infiltrate in the dermis (arrow) (haematoxylin and eosin stain, 100×). (b) Scattered, ill-formed epithelioid cell granulomas. (c) Lymphohistiocytic inflammation admixed with many neutrophils, eosinophils and Langerhans-type giant cells (arrows) (haematoxylin and eosin stain, 400×). (d) Ziehl–Neelsen stain showing acid-fast bacilli (arrow) (Ziehl–Neelsen stain, 1000×).

Biopsy of the skin revealed many scattered, ill-formed epithelioid cell granulomas and Langerhans-type giant cells, and periappendageal and perivascular lymphohistiocytic infiltrate admixed with neutrophils and eosinophils. Ziehl–Neelsen stain for acid-fast bacilli was positive (Figure 3). The overall features were suggestive of tubercular granuloma.

The patient was started on weight-based standard antitubercular therapy with four drugs (isoniazid 200 mg OD, rifampicin 400 mg OD, pyrazinamide 1 g OD and ethambutol 800 mg OD). On follow-up visits, the patient showed good clinical response—fever and pain had subsided, appetite had improved and there was gain in weight. Liver function tests normalized and erythrocyte sedimentation rate decreased to <20 mm in the first hour. A CT scan of the abdomen revealed significant decrease in the size of the periportal and peripancreatic lymph nodes. There was no evidence of biliary dilatation. The patient was doing clinically well after 6 months of antitubercular therapy.

## Discussion

Our case highlights a rare complication of ultrasound-guided FNAC, which resulted in cutaneous implantation of tubercular bacilli and incited a local granuloma. Persistent pain and formation of a nodule at the site of previous FNAC drew the attention of the patient and clinician towards the possibility of implantation along the needle tract. Biopsy of the skin nodule helped in establishing the diagnosis of tuberculosis.

Lymph nodal involvement is common in tuberculosis and access to lymph nodal tissue is necessary for obtaining the tissue. Tissue diagnosis remains the mainstay in the diagnosis and management of cases of extrapulmonary tuberculosis. FNAC is an efficient and accurate diagnostic tool for patients presenting with lymphadenopathy.^5^ FNAC is regarded as the standard of care in the diagnosis of lymph nodal tuberculosis. It is rarely associated with significant complications and has a high diagnostic yield, especially when it is image-guided. Ultrasound guidance has the advantage of real-time imaging of the needle tip during aspiration and is thus particularly suitable for fine needle aspiration (FNA) of smaller lesions.

Needle tract seeding/implantation is an iatrogenic phenomenon, typically described in relation to malignant conditions. The possible factors responsible for needle tract seeding are the use of wide-bore needle, multiple passes, superficial tumour/scanty normal parenchyma along the needle tract, high-grade tumour, large tumour size, use of the same needle for second or multiple passes and loss of suction during withdrawal of the needle. Certain sampling technique tips have recently been published that are worth following to avoid such complications.^6^ While obtaining an FNA sample, a back-and-forth motion should be used within the target lesion. Sampling should be stopped at the first sign of blood in the hub of the needle. When on-site cytology is used, the FNA sample should be obtained without aspiration using a syringe. Capillary action preferentially draws in the sample passively, whereas active aspiration typically introduces more blood into the sample and obscures the diagnostic tissue, making on-site interpretation more difficult. When FNA is used to produce a cell block or for flow cytometry, active aspiration can be used to maximize material acquisition with each pass. In our index patient, loss of suction during withdrawal of the needle and using the same needle for the second pass were the possible causes of needle tract seeding. It has rarely been defined in benign conditions such as tuberculosis.^7^ In the present case, a high bacillary load might also have contributed to needle tract seeding, as the patient was HIV-positive and tubercular lymph nodes in such patients usually have very high bacillary counts.

There is only one reported case in published literature with similar complications.^7^ The authors described a patient with thyroid swelling in whom following FNAC, the thyroid swelling increased in size and became painful, followed by development of fever. The site of FNAC needle insertion did not heal for nearly 2 months. Subsequently, the patient developed multiple small lymph nodes in the neck. The patient was operated on subsequently and histological examination revealed a sinus tract lined by tubercular granuloma. Adjacent thyroid gland and lymph nodes showed granulomatous thyroiditis. Implantation of tuberculosis bacilli along the needle tract resulted in the non-healing sinus tract following FNAC in this patient.

Tuberculosis is common in patients with HIV infection, especially in highly endemic areas for tuberculosis such as India. With the emergence of acquired immunodeficiency syndrome, the overall incidence of tuberculosis has increased. Also, there has been a shift in the presentation of tuberculosis from being predominantly pulmonary to being extrapulmonary lymph nodal tuberculosis. A high degree of suspicion is necessary in such situations, as they pose a diagnostic challenge. Mantoux test may be negative in patients with HIV–acquired immunodeficiency syndrome as there may be immune dysfunction. However, in our index case, the Mantoux test was positive, which was owing to the patient's relatively preserved CD4 count.

Our case highlights the potential for needle tract seeding during ultrasound-guided FNA in benign conditions such as tuberculosis. Presentation may be atypical and often with extrapulmonary lymph nodal involvement. FNAC, especially if image-guided, has high yield and is a very useful technique to establish diagnosis. Although FNAC is overall very safe, it can rarely be associated with complications such as implantation tuberculosis. Loss of suction during withdrawal of the needle or using the same needle for second pass during the procedure may result in needle tract seeding. Caution should be exercised to avoid needle tract seeding in benign as well as malignant conditions. This complication was a blessing in disguise for our patient, as it helped us in quickly establishing the diagnosis and starting treatment.

## Learning points

1. Needle tract seeding is a well-known complication in the setting of malignancy.
2. It can occur rarely in the setting of benign infectious conditions also.
3. Seeding of tubercular bacilli resulted in implantation tuberculosis at the skin site of needle introduction during FNAC in our index patient.
4. The risk of seeding can be reduced by using safe practices such as avoiding suction, avoiding using the same needle for second pass, minimizing the number of passes and using coaxial devices.
5. Caution should be exercised to avoid needle tract seeding even in benign conditions.

## Consent

Informed consent has been obtained from the patient and kept on record.
